# Blast Exposure Causes Long-Term Degeneration of Neuronal Cytoskeletal Elements in the Cochlear Nucleus: A Potential Mechanism for Chronic Auditory Dysfunctions

**DOI:** 10.3389/fneur.2021.652190

**Published:** 2021-03-25

**Authors:** Peethambaran Arun, Franco Rossetti, Donna M. Wilder, Ying Wang, Irene D. Gist, Joseph B. Long

**Affiliations:** Blast-Induced Neurotrauma Branch, Center for Military Psychiatry and Neuroscience, Walter Reed Army Institute of Research, Silver Spring, MD, United States

**Keywords:** blast exposure, cochlear nucleus, tinnitus, auditory dysfunction, degeneration of neuronal cytoskeletal elements

## Abstract

Blast-induced auditory dysfunctions including tinnitus are the most prevalent disabilities in service members returning from recent combat operations. Most of the previous studies were focused on the effect of blast exposure on the peripheral auditory system and not much on the central auditory signal-processing regions in the brain. In the current study, we have exposed rats to single and tightly coupled repeated blasts and examined the degeneration of neuronal cytoskeletal elements using silver staining in the central auditory signal-processing regions in the brain at 24 h, 14 days, 1 month, 6 months, and 1 year. The brain regions evaluated include cochlear nucleus, lateral lemniscus, inferior colliculus, medial geniculate nucleus, and auditory cortex. The results obtained indicated that a significant increase in degeneration of neuronal cytoskeletal elements was observed only in the left and right cochlear nucleus. A significant increase in degeneration of neuronal cytoskeletal elements was observed in the cochlear nucleus at 24 h and persisted through 1 year, suggesting acute and chronic neuronal degeneration after blast exposure. No statistically significant differences were observed between single and repeated blasts. The localized degeneration of neuronal cytoskeletal elements in the cochlear nucleus suggests that the damage could be caused by transmission of blast shockwaves/noise through the ear canal and that use of suitable ear protection devices can protect against acute and chronic central auditory signal processing defects including tinnitus after blast exposure.

## Introduction

Hearing loss and tinnitus are among the most prevalent disabilities reported in veterans, and the incidence of hearing deficits in service members greatly exceeds that of the civilian population in the United States (1). Although auditory system damage from gunfire or weapon systems (e.g., artillery, mortars) has historically been the predominant cause of combat-related hearing loss, blast. exposure is another major cause of auditory system damage and its incidence has risen sharply in recent military conflicts as a result of the widespread use of improvised explosive devices. Up to 62% of blast-injured patients exhibit hearing loss and tinnitus (2). Hoffer et al. (3) evaluated Marines with mild traumatic brain injury (mTBI) from combat-related blasts and found that the prevalence of hearing loss was 33% in acute patients and 49% in chronic patients. The total annual expense to deliver healthcare services to treat hearing dysfunctions and compensate veterans for hearing impairment has been estimated to exceed $1 billion (4). From 2006 to 2010, the number of veterans who received new compensation for impairment of auditory acuity grew by more than 72% (5).

Although hearing personal protective equipment (PPE) is available in the field operations, anecdotal reports indicate that some troops decline to wear hearing protection due to fear of reduced situational awareness in the battlefield, which increases the incidence of blast-induced hearing loss and tinnitus (5, 6). Improper use of the hearing PPE also causes injury to the auditory system after blast exposure. Blast-induced injuries to the ear often present as damage to the sensitive structures of the inner and middle ear, such as the cochlea, ossicular chain, tympanic membrane (TM), and vestibular system (7–9). Ruptured TM is one of the common acute pathological features after blast exposure, and the severity increases with the intensity of blast. Personnel with TM rupture after blast exposure have higher incidence of hearing loss and tinnitus outcomes than those without TM rupture (5). Long-term sensorineural hearing loss has been reported in patients with TM perforations after blast exposure (10). In laboratory rats, we have observed ruptured TM at 1 and 7 days post-blast, and the healing of the TM occurred at 1 month without any treatments (9). In many victims of blast exposure, severe auditory dysfunctions occur despite an intact TM, suggesting that hearing loss can result from both inner ear injury, and central auditory signal processing defects (CASPD) (11). It is still uncertain whether those changes in hearing are due to injury to the central or peripheral auditory system or injury to both.

Preclinical studies using different animal models and blast simulations indicated that blast exposure causes acute and chronic hearing impairments including tinnitus. Multiple pathophysiological mechanisms responsible for blast-induced auditory dysfunctions have been proposed in experimental subjects using different blast simulations (12–15). Most preclinical studies have focused on the injuries to the outer, middle, and inner ears after blast exposure (9, 12–15). Recently, we have shown that tightly coupled repeated blasts lead to acute and subacute pathological changes in the inner ear resulting in impaired auditory functions measured using Auditory Brainstem Response (ABR) and Distortion Product Otoacoustic Emission (DPOAE) (9). Genomic analysis of the inner ear samples on days 1 and 28 post-blast exposures revealed differential expression of several genes involved in mechanotransduction, cytoskeletal reorganization, myelin development, and axon survival (9). Few preclinical studies have explored the acute and subacute changes in auditory signal processing centers in the brain after blast exposure (13, 16, 17). In the present study, we have evaluated the effect of single and repeated blasts on neuronal degeneration in the brain regions involved in auditory signal processing in rats to determine whether blast exposure can cause acute and chronic CASPD.

## Materials and Methods

### Animals

All animal experiments were conducted in accordance with the Animal Welfare Act and other federal statutes and regulations relating to animals and experiments involving animals and adhered to principles stated in the Guide for the Care and Use of Laboratory Animals (NRC Publication 2011 edition) using an Institutional Animal Care and Use Committee approved protocol. Male Sprague Dawley rats, 9–10 weeks old weighing 300–350 g (Charles River Laboratories, Wilmington, MA), were housed at 20–22°C (12 h light/dark cycle). Rats were given free access to nutritious rat chow (Prolab IsoPro RMH 3,000 from LabDiet, St. Louis, MO) and water *ad libitum* till 1 month after the blast exposure, when they reached a body weight of 400–450 g. We restricted diet for all rats including sham controls after 1 month so that the weight of the rats was maintained between 450 and 500 g until the completion of the study (1 year). Body weights were recorded 3 days a week and adjustments were made in the quantity of diet to maintain body weights within this range since these animals were also used for neurobehavioral functional tests summarized in a recent publication (18).

### Primary Blast Exposure

The advanced blast simulator (ABS) described previously was used for the blast exposure (18, 19). For blast exposure, the rats were anesthetized with 4% isoflurane for 8 min and secured in a longitudinal (i.e., rat facing the oncoming shockwave) prone orientation in the test section of the ABS. To produce moderate injury to the auditory system, we used Valmax membranes yielding peak positive static pressures of ~19 psi with a positive phase duration of 4–5 msec. For tightly coupled repeated blast exposures, the rats were exposed to two 19-psi blast overpressure waves separated by 2 min as described earlier (18, 19). Sham control rats received only the anesthesia and handling and were not exposed to blast. Four rats were used for sham controls, and six rats each were used for single and repeated blast-exposed groups for different time points. After blast exposure, the rats were euthanized at 24 h, 14 days, 1 month, 6 months, or 1 year.

### Silver Staining

At each time point after blast exposure, the animals were anesthetized by inhalationally administering 5 % isoflurane for 6 min and then were transcardially perfused first with normal saline followed by 4% paraformaldehyde containing 0.15% picric acid. The brains were collected and post-fixed in 4% paraformaldehyde for 6 h followed by cryopreservation using 20% sucrose solution. The brains were sent to FD Neurotechnologies (Columbia, MD) for silver staining using their Neurosilver Kit II in 50-μm coronal sections with 400-μm intervals between them to span all auditory signal-processing regions evaluated in the brain. All the five brain regions from the three treatment groups for a particular time point were processed and stained at the same time. Because of batch-to-batch variation of staining intensities, longitudinal comparisons were not made across time points for any of the treatment groups. This staining technique is based on the finding that neuronal axons, somata, and terminals undergoing degeneration become particularly argyrophilic and, under these conditions, these cytoskeletal elements bind silver ions with high affinity. Upon reduction, the silver ions form metallic grains that are visible under a light microscope and can be quantitated by densitometry. The staining was performed at FD Neurotechnologies according to the protocol developed by the company and described in their kit instructions. Sections were mounted on to glass slides for microscopic evaluations. The different brain regions involved in central auditory signal processing were selected using the rat brain atlas of Paxinos and Watson (20). Since we evaluated three 50-μm sections with 400 μm separating them, there was potential overlap with an adjacent cell layer. Such interferences were minimized by carefully following the rat brain atlas. Photographs of different silver-stained brain regions were taken using an Olympus BX61 microscope (Olympus Corporation, Center Valley, PA) and Stereo Investigator virtual image tool (MBF Biosciences, Williston, VT) connected to a computer. After all pictures were taken, the regions to be analyzed were selected and their outlines traced in the respective pictures (three adjacent slices per rat for each region with a 400-μm interval between them), using the Select Region Tool option of Image-Pro Premier 9.2 software (Media Cybernetics Inc, Rockville, MD) to inform the software where the densitometry needs to be performed. Using the Count/Size tool and Manual Threshold Tool, we defined through monochromatic histogram a specific range of black color that masks only black-colored spots. Subsequently, a black and white image mask was made from the previous image where black grains marked in the original picture appear in white and background in black. The densitometry analysis was applied in the image mask, and the software automatically calculated the density of white regions as Integrated Optical Densities (lum/mm^2^). Some minor artifacts during the staining process also appear as small black spots which the program recognized and counted along with the black spots from the silver reduction. Since these artifacts were present in all treatment groups including sham controls, a comparison between the three treatment groups was carried out to minimize this confounding variability.

### Statistical Analysis

Statistical analysis was carried out by one-way analysis of variance (ANOVA) followed by Holm–Sidak *post-hoc* test (SigmaPlot 12.5 software). All blast-exposed animals were compared with a respective sham control group. The repeated blast-exposed animals were compared to the single blast-exposed animals. Values were expressed as mean ± standard error of the mean (SEM). A *p*-value < 0.05 was considered significant.

## Results

### Silver Staining in the Auditory Cortex

**Figure 1:**
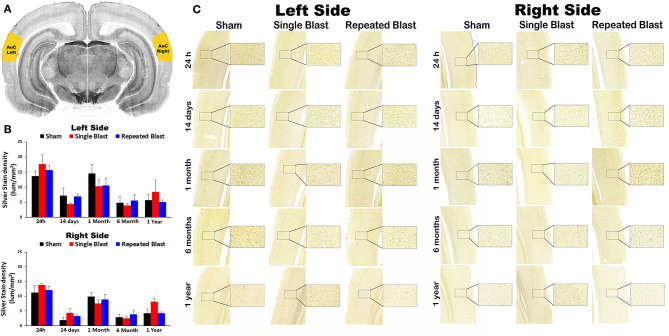
Silver staining in the left and right auditory cortex. **(A)** The auditory cortex (AuC) region selected using the rat brain atlas for analysis. **(B)** Graphics showing densitometry analysis from the left and right silver-stained AuC regions at different intervals post-blast exposures. **(C)** Representative silver-stained images from the left and right AuC regions at different intervals after single and tightly coupled repeated blasts. Portions of sections inside rectangles are shown at higher magnification on the right. Values are expressed as mean ± SEM. Density values of single and repeated blast-exposed groups were compared to the respective sham controls (*p* > 0.05, ANOVA; normal distribution; *n* = 4–6).

Silver staining in the left and right auditory cortex regions at 24 h, 14 days, 1 month, 6 months, and 1 year showed no statistically significant changes (*p*-values were >0.05) in degeneration of neuronal cytoskeletal elements after blast exposure compared to sham controls (Figure 1). In the right hemisphere, silver-stain densities for sham controls, and single and repeated blast-exposed animals were 11.27 ± 2.33, 13.86 ± 0.47, and 12.13 ± 1.21, respectively at 24 h; 1.97 ± 0.84, 4.36 ± 1.44, and 3.32 ± 0.42, respectively at 14 days; 9.86 ± 1.42, 7.53 ± 1.29, and 8.93 ± 1.80, respectively at 1 month; 2.90 ± 0.88, 2.51 ± 0.82, and 3.86 ± 1.27, respectively at 6 months; and 4.26 ± 1.40, 8.17 ± 2.44, and 4.32 ± 0.42, respectively at 1 year. In the left hemisphere, silver-stain densities for sham controls and single and repeated blast-exposed animals were 13.65 ± 1.72, 17.63 ± 3.05, and 15.64 ± 1.48, respectively at 24 h; 7.12 ± 2.58, 4.50 ±0.32, and 6.92 ± 0.82, respectively at 14 days; 14.60 ± 2.98, 10.28 ± 2.28, and 10.52 ± 2.50, respectively at 1 month; 4.86 ± 2.03, 4.01 ± 0.66, and 5.61 ± 2.08, respectively at 6 months; and 5.69 ± 1.88, 8.49 ± 3.89, and 5.12 ± 0.61, respectively at 1 year. No statistically significant changes (*p*-values were >0.05) in degeneration of neuronal cytoskeletal elements in the auditory cortex were observed between single and repeated blast-exposed animals at any of the time points evaluated.

### Silver Staining in the Medial Geniculate Nucleus

**Figure 2:**
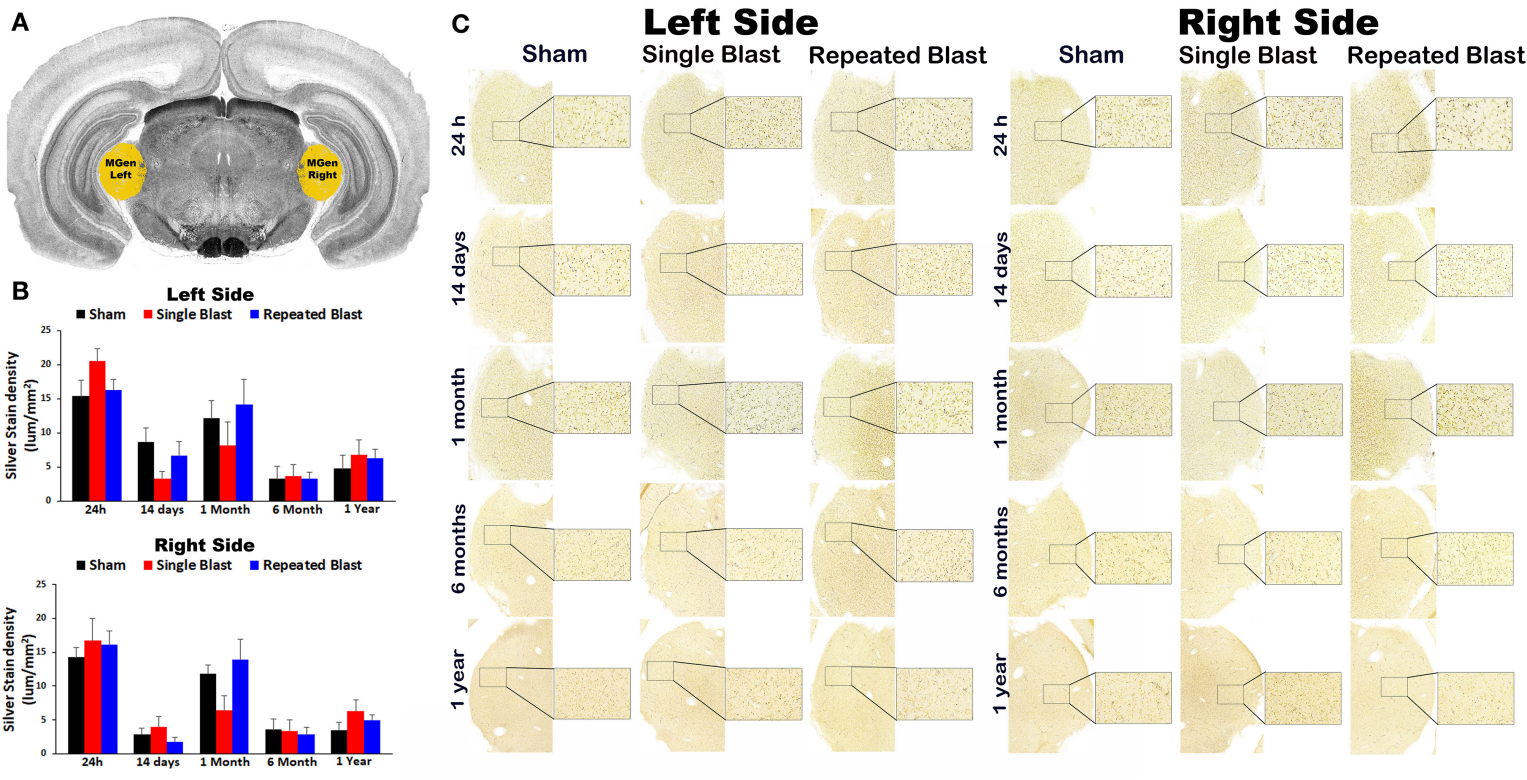
Silver staining in the left and right medial geniculate nucleus. **(A)** Medial geniculate (MGen) region selected using rat brain atlas for analysis. **(B)** Graphics showing densitometry analysis from the left and right silver-stained MGen regions at different intervals post-blast exposures. **(C)** Representative silver-stained images from left and right MGen regions at different intervals after single and tightly coupled repeated blasts. Portions of sections inside rectangles are shown at higher magnification on the right. Values are expressed as mean ± SEM. Density values of single and repeated blast-exposed groups were compared to the respective sham controls (*p* > 0.05, ANOVA; normal distribution; *n* = 4–6).

Compared to sham control animals, silver staining did not show any statistically significant differences (*p*-values were >0.05) in degenerated neuronal cytoskeletal elements in the medial geniculate nucleus on the left and right sides at any of the time points evaluated after blast exposure (Figure 2). On the right side, silver-stain densities for sham controls and single and repeated blast-exposed animals were 14.26 ± 3.23, 16.68 ± 1.47, and 16.09 ± 2.05, respectively at 24 h; 2.89 ± 0.85, 3.97 ± 1.48, and 1.74 ± 0.66, respectively at 14 days; 11.82 ± 1.27, 6.36 ± 2.17, and 13.87 ± 3.08, respectively at 1 month; 3.52 ± 1.55, 3.34 ± 1.67, and 2.83 ± 1.07, respectively at 6 months; and 3.46 ± 1.19, 6.24 ± 1.74, and 4.98 ± 0.72, respectively at 1 year. On the left side, silver-stain densities for sham controls and single and repeated blast-exposed animals were 15.41 ± 2.35, 20.51 ± 1.90, and 16.32 ± 1.50, respectively at 24 h; 8.71 ± 2.01, 3.34 ± 1.03, and 6.65 ± 2.12, respectively at 14 days; 12.16 ± 2.51, 8.22 ± 3.44, and 14.23 ± 3.64, respectively at 1 month; 3.25 ± 1.86, 3.61 ± 1.78, and 3.26 ± 0.91, respectively at 6 months; and 4.77 ± 1.90, 6.77 ± 2.25, and 6.36 ± 1.28, respectively at 1 year. No statistically significant changes in degenerated neuronal cytoskeletal elements were observed (*p*-values were >0.05) in this brain region between single and repeated blast-exposed rats.

### Silver Staining in the Lateral Lemniscus

**Figure 3:**
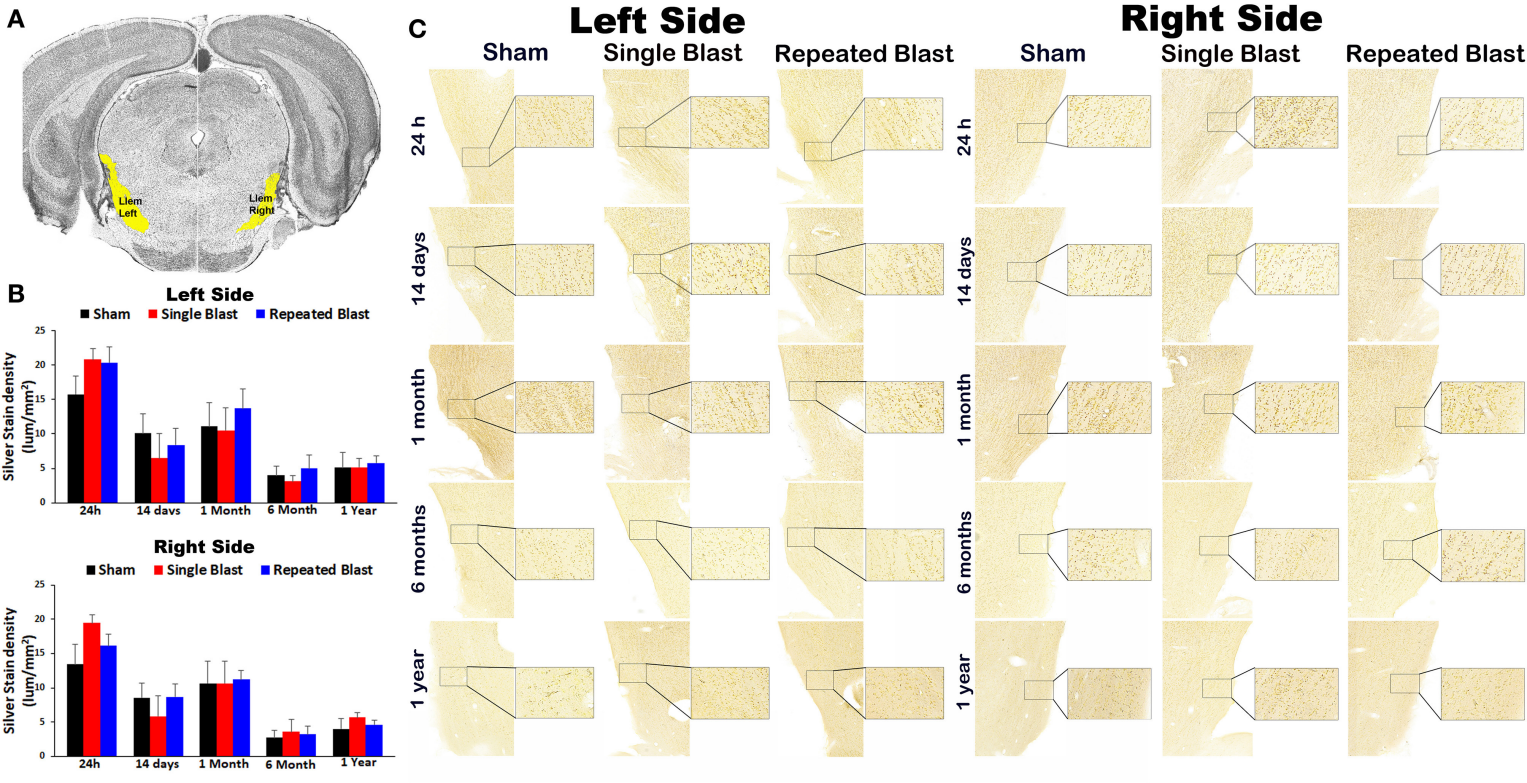
Silver staining in the left and right lateral lemniscus. **(A)** Lateral lemniscus (Llem) region selected using the rat brain atlas for analysis. **(B)** Graphics showing densitometry analysis from the left and right silver-stained Llem regions at different intervals post-blast exposures. **(C)** Representative silver-stained images from the left and right Llem regions at different intervals after single and tightly coupled repeated blasts. Portions of sections inside rectangles are shown at higher magnification on the right. Values are expressed as mean ± SEM. Density values of single and repeated blast-exposed groups were compared to the respective sham controls (*p* > 0.05, ANOVA; normal distribution; *n* = 4–6).

Figure 3 shows silver staining in the lateral lemniscus region in the brainstem. On the right side, silver stain densities for sham controls and single and repeated blast-exposed animals were 13.38 ± 2.92, 19.44 ± 1.25, and 16.10 ± 1.72, respectively at 24 h; 8.57 ± 2.07, 5.87 ± 2.93, and 8.68 ± 1.87, respectively at 14 days; 10.63 ± 3.29, 10.66 ± 3.27, and 11.18 ± 1.36, respectively at 1 month; 2.72 ± 1.00, 3.54 ± 1.82, and 3.21 ± 1.13, respectively at 6 months; and 3.96 ± 1.54, 5.62 ± 0.73, and 4.61 ± 0.66, respectively at 1 year. On the left side, silver stain densities for sham controls and single and repeated blast-exposed animals were 15.75 ± 2.60, 20.79 ± 1.54, and 20.33 ± 2.34, respectively at 24 h; 10.12 ± 2.82, 6.44 ± 3.61, and 8.40 ± 2.44, respectively at 14 days; 11.11 ± 3.44, 10.50 ± 3.32, and 13.67 ± 2.90, respectively at 1 month; 4.00 ± 1.34, 3.09 ± 0.87, and 4.97 ± 1.97, respectively at 6 months; 5.12 ± 2.17, 5.15 ± 1.28, and 5.80 ± 0.98, respectively at 1 year. No statistically significant changes in silver-stained neuronal cytoskeletal elements were observed (*p*-values were >0.05) in the left and right lateral lemniscus regions at any of the time points evaluated after blast exposure compared to sham controls. Compared to single blast-exposed rats, no significant changes (*p*-values were >0.05) in degenerated neuronal cytoskeletal elements were observed in the repeated blast-exposed rats in this brain region involved in auditory signal processing.

### Silver Staining in the Inferior Colliculus

**Figure 4:**
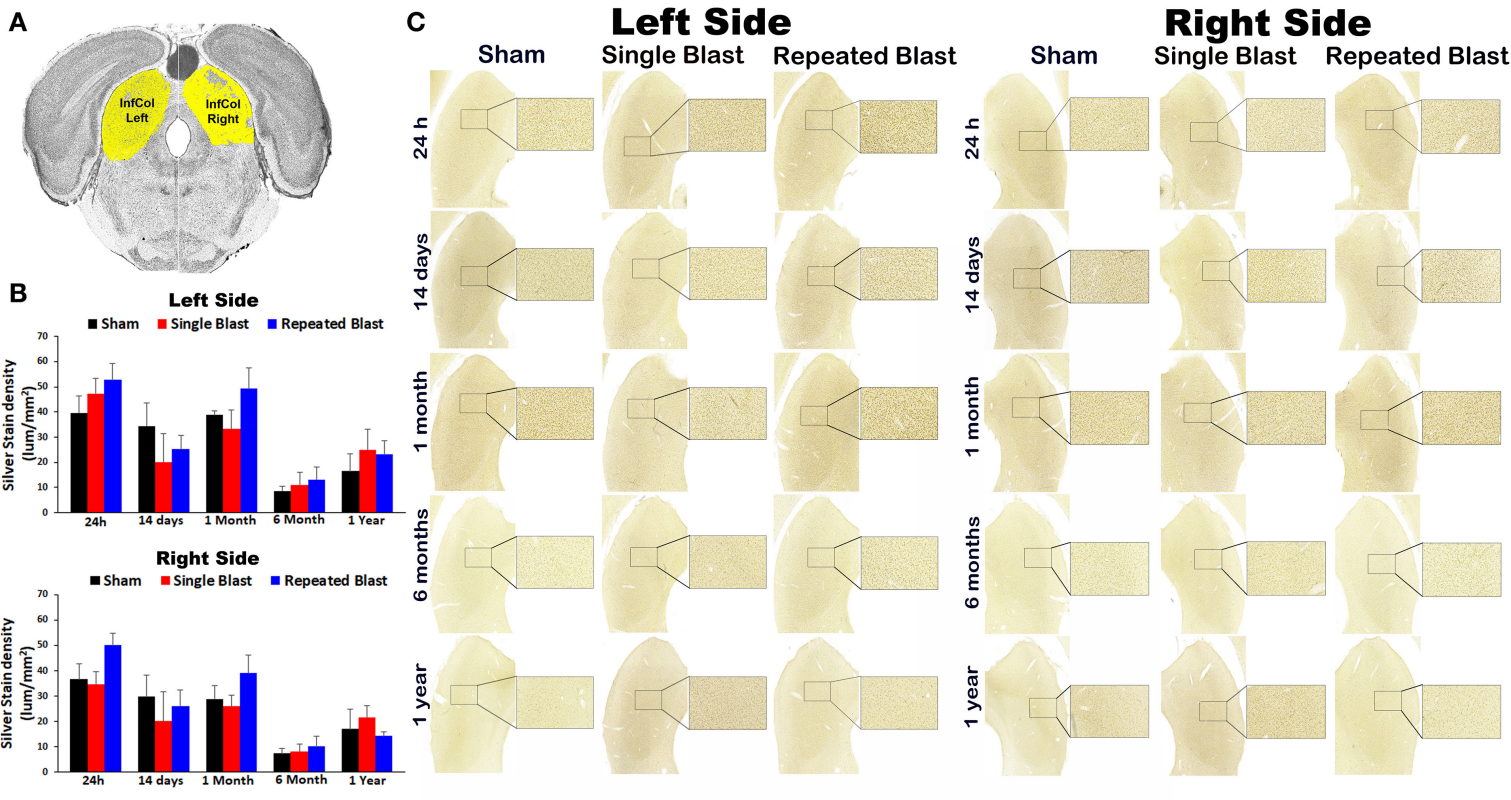
Silver staining in the left and right inferior colliculus. **(A)** Inferior colliculus (InfCol) region selected using rat brain atlas for analysis. **(B)** Graphics showing densitometry analysis from the left and right silver-stained InfCol regions at different intervals post-blast exposures. **(C)** Representative silver-stained images from left and right InfCol regions at different intervals after single and tightly coupled repeated blasts. Portions of sections inside rectangles are shown at higher magnification on the right. Values are expressed as mean ± SEM. Density values of single and repeated blast-exposed groups were compared to the respective sham controls (*p* > 0.05, ANOVA; normal distribution; *n* = 4–6).

Silver staining in the left and right inferior colliculus regions revealed no statistically significant changes (*p*-values were >0.05) in degenerated neuronal cytoskeletal elements at any of the time points evaluated after blast exposure compared to sham controls (Figure 4). On the right side, silver-stain densities for sham controls and single and repeated blast-exposed animals were 36.57 ± 6.09, 34.48 ± 5.16, and 50.01 ± 4.61, respectively at 24 h; 29.65 ± 8.73, 20.09 ± 11.56, and 26.17 ± 6.15, respectively at 14 days; 28.82 ± 5.39, 25.92 ± 4.52, and 39.03 ± 7.29, respectively at 1 month; 7.33 ± 2.01, 7.98 ± 3.22, and 10.06 ± 4.03, respectively at 6 months; 17.01 ± 793, 21.59 ± 4.54, and 14.30 ± 1.63, respectively at 1 year. On the left side, silver-stain densities for sham controls, single, and repeated blast-exposed animals were 39.59 ± 6.69, 47.30 ± 5.90, and 52.77 ± 6.61, respectively at 24 h; 34.44 ± 9.12, 20.25 ± 11.06, and 25.20 ± 5.51, respectively at 14 days; 38.78 ± 1.84, 33.22 ± 7.60, and 49.23 ± 8.18, respectively at 1 month; 8.72 ± 1.91, 10.93 ± 5.09, and 13.19 ± 5.10, respectively at 6 months; and 16.59 ± 6.83, 24.93 ± 8.22, and 23.07 ± 5.39, respectively at 1 year. Similar to the above brain regions involved in auditory signal processing, no statistically significant (*p*-values were >0.05) in degenerated neuronal cytoskeletal elements were observed in the left and right inferior colliculus regions of single and repeated blast-exposed rats.

### Silver Staining in the Cochlear Nucleus

**Figure 5:**
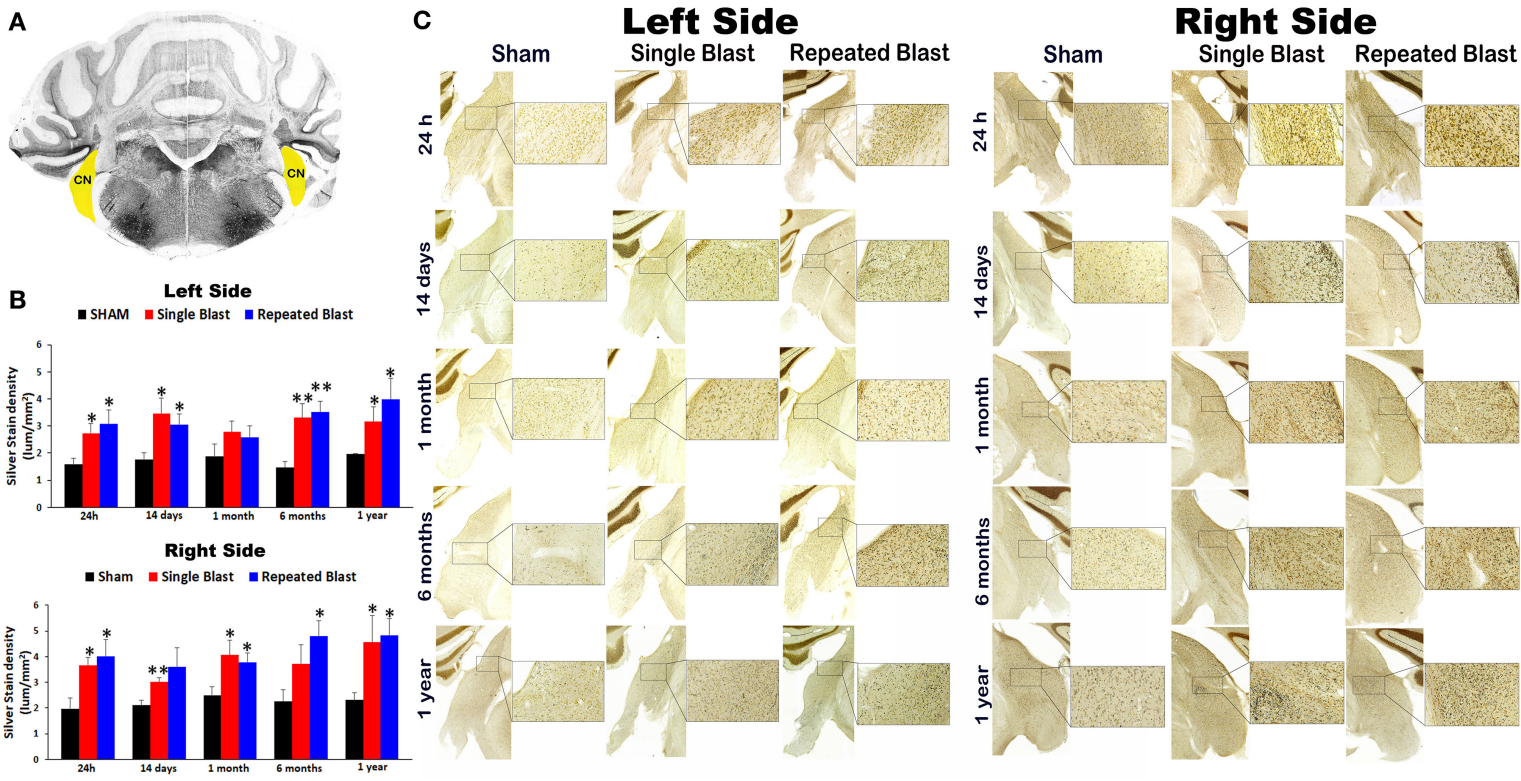
Silver staining in the left and right cochlear nuclei. **(A)** Cerebellum and brainstem structures showing cochlear nucleus (CN) selected using the rat brain atlas for analysis. **(B)** Graphics showing densitometry analysis from left and right silver-stained CN at different intervals post-blast exposures. **(C)** Representative silver-stained images from left and right CN at different intervals after single and tightly coupled repeated blasts. Portions of sections inside rectangles are shown at higher magnification on the right. Values are expressed as mean ± SEM. Density values of single and repeated blast-exposed groups were compared to the respective sham controls (**p < 0.01; *p < 0.05; ANOVA; normal distribution; *n* = 4–6).

Silver staining of the left side revealed acute and chronic degeneration of neuronal cytoskeletal elements in the left cochlear nucleus (CN) at 24 h, 14 days, 6 months, and 1 year after single and tightly coupled repeated blasts (Figure 5). On the left side, silver-stain densities for sham controls and single and repeated blast-exposed animals were 1.58 ± 0.23, 2.73 ± 0.34, and 3.06 ± 0.52, respectively at 24 h (*p* values for single and repeated blast-exposed groups compared to sham controls were 0.019 and 0.03, respectively); 1.77 ± 0.23, 3.46 ± 0.55, and 3.04 ± 0.40, respectively at 14 days (*p*-values for single and repeated blast-exposed groups compared to sham controls were 0.019 and 0.023, respectively); 1.87 ± 0.46, 2.77 ± 0.40, and 2.57 ± 0.44, respectively at 1 month (*p*-values for single and repeated blast-exposed groups compared to sham controls were >0.05); 1.47 ± 0.22, 3.31 ± 0.50, and 3.52 ± 0.38, respectively at 6 months (*p*-values for single and repeated blast-exposed groups compared to sham controls were 0.009 and 0.006, respectively); 1.95 ± 0.02, 3.16 ± 0.54, and 3.99 ± 0.76, respectively at 1 year (*p*-values for single and repeated blast-exposed groups compared to sham controls were 0.035 and 0.025, respectively). Blast exposure caused acute and chronic degeneration of neuronal cytoskeletal elements in the CN of the right side at all the time points evaluated (Figure 5). On the right side, silver-stain densities for sham controls and single and repeated blast-exposed animals were 1.95 ± 0.42, 3.66 ± 0.30, and 4.02 ± 0.66, respectively at 24 h (the *p*-value for single and repeated blast-exposed groups compared to sham controls was 0.023); 2.10 ± 0.20, 3.01 ± 0.17, and 3.61 ± 0.71, respectively at 14 days (the *p*-value for the single blast-exposed group compared to sham controls was 0.01 whereas that for the repeated blast-exposed group was >0.05); 2.48 ± 0.35, 4.05 ± 0.57, and 3.77 ± 0.37, respectively at 1 month (*p*-values for single and repeated blast-exposed groups compared to sham controls were 0.047 and 0.049, respectively); 2.26 ± 0.43, 3.72 ± 0.73, and 4.79 ± 0.60, respectively at 6 months (the *p*-value for the single blast-exposed group compared to sham controls was >0.05 whereas that for the repeated blast-exposed group was 0.026); and 2.31 ± 0.26, 4.56 ± 1.05, and 4.83 ± 0.63, respectively at 1 year (*p*-values for single and repeated blast-exposed groups compared to sham controls were 0.04 and 0.035, respectively). No statistically significant changes in degenerated neuronal cytoskeletal elements were observed between the single and repeated blast-exposed groups at all the time points evaluated in the left and right sides (*p*-values were >0.05).

## Discussion

Although auditory dysfunction including tinnitus is one of the major disabilities affecting military personnel after the increased use of improvised explosive devices, the precise pathological mechanism(s) is still not identified, which has hampered the development of effective countermeasures. Preclinical studies in mice have shown that blast exposure disrupted the TM; healing of the membrane occurred within a few days after the exposure, although the healed TM was thickened (9, 14). Auditory functional tests indicated that the hearing deficits were more severe in the blast-exposed animals in comparison to those animals simply subjected to surgical perforation of the TM, suggesting that the blast injury yielded additional damage to the inner ear structures and/or the injury to the central auditory signal processing regions (14). Auditory Brainstem Response (ABR) and Distortion Product Otoacoustic Emission (DPOAE) threshold shifts were correlated with blast intensity and were associated with loss of outer hair cells within the basal turn of the cochlea along with decreased spiral ganglion neurons and afferent nerve synapses (14). Five weeks after a 14-psi blast exposure, 8 out of 13 rats exhibited chronic tinnitus; all exposed rats showed sustained ABR wave amplitude reduction, whereas no changes in acoustic pre-pulse inhibition or ABR thresholds were observed (21). Exposure of rats to 22-psi blasts led to changes in spontaneous firing rates in the dorsal CN and spontaneous activity changes in the inferior colliculus, suggesting CASPD after blast exposure (17, 22). Recently, using an ABS, which generates blast waves very similar to free field explosions, we also have shown in experimental rats that blast exposure disrupted the TM and affected auditory function at acute and subacute time points after tightly coupled repeated blast exposures with a 16-psi blast overpressure (9). ABR thresholds increased significantly over all tested frequencies from 4 to 40 kHz immediately after blast exposure at 16 psi and persisted for 28 days (9). The earliest improvement in the ABR threshold was noted at 7 days after injury in the 8–11.2-kHz frequency range (9). On day 28 post-blast at 16 psi, the shifted ABR thresholds were recovered partially at 32 kHz, but no improvement was observed at 40 kHz (9). Abnormal DPOAE signals were observed from day 1 and persisted until 28 days, suggesting defective outer hair cells after blast exposure (9). Loss of outer hair cells after blast exposure was reported previously in experimental animals (14). To understand the mechanism of hearing loss after blast exposure and the role of pathological changes occurring in the inner ear, we have explored the differential expression of genes in the inner ear and found changes in different genes potentially involved in hearing dysfunctions after blast exposure (9). Some of the differentially expressed genes in the inner ear samples on days 1 and 28 post-blast are reported to be involved in mechanotransduction, cytoskeletal reorganization, myelin development, and axon survival (9). No prior studies have established changes occurring in the central auditory signal processing regions in the brain after single or repeated blasts using an ABS or similar blast simulation device.

In the current study, using an ABS, we have shown for the first time that single and repeated blast exposures lead to acute and chronic degeneration of neuronal cytoskeletal elements in the CN (Figure 5). The silver impregnation method we adopted has been commonly used for identifying neuron populations undergoing programmed cell death in the developing brain (23). In addition, the neuronal debris formed after apoptotic or necrotic cell death also can be stained with the technique, but the method cannot distinguish which cell death pathway has occurred (23). The silver impregnation occurs mostly in the neuronal cytoskeletal elements such as axons, lysosomes, and neuronal terminals. Sustained degeneration of neuronal cytoskeletal elements was observed in the CN of both sides from 24 h to 1 year post-blast. Interestingly, no statistically significant differences in the magnitudes of degenerated neuronal cytoskeletal elements were observed in the left or right CN of single and tightly coupled repeated blast-exposed animals at any of the time points evaluated. Damage to neurons in the CN, especially dorsal CN, has been reported to cause acute and chronic auditory dysfunctions including tinnitus (24, 25). Using electron microscopy, it has been shown that noise exposure unaccompanied by blast overpressure causes long-term neurodegeneration in the CN with freshly occurring degeneration of neurons as late as 8 months (25). A single noise exposure resulted in loss of cell density in different central auditory signal processing regions, including CN, at 1 week post-exposure (26). Noise exposure caused differential expression of pro- and anti-apoptotic genes in the CN and other central auditory signal processing regions, suggesting an acute intrinsic apoptosis process in those brain regions after noise trauma (27, 28). Acoustic insult in rats caused neurodegeneration in the anteroventral CN (AVCN) without changes in the posteroventral and dorsal CN (PVCN and DCN) at 9 weeks post-injury (29). In contrast, mechanical compression resulted in loss of neurons in the PVCN and DCN and not in the AVCN, revealing that different types of insults cause different types of injuries to the central auditory signal processing system (29, 30). In the current study using blast exposure, the injury to the CN could result from a combination of insults associated with the transmission of noise as well as blast over pressure waves through the ear canal, resulting in unique acute and chronic changes in the CN. The sustained degeneration of neuronal cytoskeletal elements observed until 1 year could be due to progressive secondary injuries to the inner ear structures extending to the CN or resulting from secondary injury mechanisms developing in the CN itself. It is interesting to note that no significant changes in degeneration of neuronal cytoskeletal elements in the CN were observed between the single and tightly coupled repeated blast-exposed rats. Similarly, the progression of apoptosis in the CN and inferior colliculus did not differ between the single and repeated noise-exposed mice and indicated that the first noise trauma had a long-lasting effect on apoptotic mechanisms in the central auditory pathway that was not substantially altered by a second trauma (31). In contrast, the same research group reported previously that the repeated noise-exposed group had increased apoptotic markers in the auditory cortex and medial geniculate body (32). Since the present study determined only degeneration of neuronal cytoskeletal elements using silver staining, it is somewhat surprising and unclear why there was no significant difference between single and tightly coupled repeated blast-exposed animals. Unraveling the precise mechanism of degeneration of neuronal cytoskeletal elements induced by blast exposure is essential for solving this mystery. In the current study, although we have not explored the apoptotic or necrotic mechanisms in the inner ear or CN, it is possible that such mechanisms may contribute to the degeneration of neuronal cytoskeletal elements in the CN after blast exposure. In this perspective, it has been shown that a single blast exposure at higher intensity led to neuronal apoptosis mediated by the caspase-dependent pathway at acute time points (33). The silver staining used in the current study can measure only the degeneration of neuronal cytoskeletal elements, but the changes in glial cells after blast exposure can indirectly play significant roles in the auditory impairments. Activation of glial cells and resulting neuroinflammation also promote neuronal degeneration (34). Future longitudinal study exploring the changes in both peripheral auditory system and CN is warranted to better identify the mediators and mechanism(s) yielding chronic degeneration of neuronal cytoskeletal elements in the CN after single and repeated blast exposures.

Silver staining in the other brain regions involved in central auditory signal processing, such as auditory cortex, medial geniculate nucleus, lateral lemniscus, and inferior colliculus, revealed no statistically significant blast-induced changes in the degenerated neuronal cytoskeletal elements at any of the time points evaluated after overpressure exposure in the ABS (Figures 1–4). Moreover, no differences in degeneration of neuronal cytoskeletal elements were observed in these regions when comparing single and repeated blast-exposed animals. As described in the methods sections, since we have used three 50-μm sections separated by 400 μm for a particular brain region, there were potential overlaps with the adjacent brain regions. The large error bars in the densitometry measurements support this potential interference, which we tried to minimize by carefully following the rat brain atlas by Paxinos and Watson (20). Analyzing large auditory structures with silver staining is suboptimal, and the findings warrant follow-up with more precise neuroanatomical descriptions. Markers of neurodegeneration such as neurofilament light chain and pathologic Tau oligomer accumulations were observed in the central (auditory cortex) and peripheral auditory systems (spiral ganglion) of rats exposed to blast using a cylindrical shock tube (35). Diffusion tensor imaging studies in rats exposed to 14-psi blasts in a cylindrical shock tube revealed significant microstructural changes in central auditory signal processing regions such as the inferior colliculus and medial geniculate body without changes in the corpus callosum (13). As indicated above, these two prior studies were carried out using cylindrical shock tubes which are reported to cause artifacts in blast conditions due to significant contribution of blast wind (36), which is minimal in the ABS used for the current study (18). The animals utilized for the current study using an ABS also underwent neurobehavioral functional assessments from 24 h to 1 year post-blast. The neurobehavioral functional assessments revealed acute functional deficits, most of which recovered toward normal during the subacute stages but deteriorated again after 6 and 12 months after single and tightly coupled repeated blasts (18). None of these neurobehavioral functional tests required auditory function; hence, the current results showing sustained degeneration of neuronal cytoskeletal elements in the CN cannot be readily, directly compared and correlated with behavioral outcomes assessed in the same population of experimental subjects.

It has been reported that blast waves transmit through the ear canal leading to disruption of tympanic membrane and inner ear structures involved in peripheral auditory system (14). Mao et al. (13) reported microstructural changes in the inferior colliculus and medial geniculate body in the brain and proposed that blast exposure affects the central auditory signaling through the peripheral auditory pathway (outer ear to inner ear to brain) and not through direct biomechanical impact on the brain parenchyma. Our current data showing localized degeneration of neuronal cytoskeletal elements in the CN after blast exposure support the above notion that blast shockwaves and noise transmit through the ear canal and reach the CN in the brainstem. Axonal degeneration is reported to play a major role in the development of chronic neuronal degeneration observed in neurodegenerative diseases and TBI (37, 38). A future longitudinal study assessing auditory functions after blast exposure is warranted to confirm whether the degeneration of neuronal cytoskeletal elements in the CN is responsible for the chronic auditory dysfunctions after blast exposure. Although it is still unknown that the localized degeneration of neuronal cytoskeletal elements we have observed after blast exposure was due to blast shockwaves or noise transmission through the ears, the proper use of suitable ear protection devices might protect against short- and long-term auditory impairments resulting from either biomechanical insult associated with blast exposure.

## Data Availability Statement

The original contributions presented in the study are included in the article/supplementary material, further inquiries can be directed to the corresponding author/s.

## Ethics Statement

The animal study was reviewed and approved by Institutional Animal Care and Use Committee, Walter Reed Army Institute of Research.

## Author Contributions

PA and JL designed the experiments. DW and IG performed the blast experiments and sample collections. FR and YW performed analyses of silver stained sections. PA and JL wrote the manuscript. All authors contributed to the article and approved the submitted version.

## Conflict of Interest

The authors declare that the research was conducted in the absence of any commercial or financial relationships that could be construed as a potential conflict of interest.
